# Effect of APPT Treatment on Mechanical Properties and Durability of Green Composites with Woven Flax

**DOI:** 10.3390/ma13214762

**Published:** 2020-10-25

**Authors:** Belén Enciso, Juana Abenojar, Miguel Angel Martínez

**Affiliations:** IAAB, Materials Performance Group, Materials Science and Engineering Department, Universidad Carlos III de Madrid, 28911 Leganés, Spain; abenojar@ing.uc3m.es (J.A.); mamc@ing.uc3m.es (M.A.M.)

**Keywords:** natural fibres, green composites, mechanical properties, surface treatments, durability

## Abstract

Through this study, two different natural fibres green composites were characterised from the point of view of mechanical properties and durability. These green polymers allow manufacturing with a respectful life cycle due to their biodegradable or recyclable character. Composite materials were prepared in a hot plates press with two biopolymeric matrices, green low density polyethylene (GPE) and polybutylene succinate (PBS). As reinforcement, Atmospheric Pressure Plasma Torch (APPT) treated and untreated unidirectional woven flax were used. Mechanical properties were evaluated by tensile tests and the adhesion between matrices and reinforcement by peeling tests. The durability of each composite was analysed by water absorption measurements, Fourier Transform Infrared Spectroscopy (FTIR) analysis and tensile tests, during several aging times, up to 60 days, under high temperature and humidity conditions. The influence of the Atmospheric Pressure Plasma Torch treatment (APPT) was evaluated in all studies. It was found that GPE composites present better durability against aging conditions than PBS materials, due to the tendency of polyester to hydrolyse compared to the good resistance to humidity of polyolefins. The adhesion between matrices and reinforcement improves with APPT treatment. This improvement is more evident by avoiding the absorption of water than in the mechanical properties results, where only a slightly improvement is shown.

## 1. Introduction

Natural fibres reinforced polymeric composites are considered an important alternative to those reinforced with synthetic fibres. Many studies are focused on its research in the last decades [1,2,3,4]. Natural fibres are biodegradable reinforcement with excellent mechanical properties, low cost, high availability and low density, allowing to obtain lighter weight environmentally friendly composites [5].

Mechanical properties of natural fibres are mainly determined by their composition [6], as the fibres are lignocellulosic structures mostly formed by cellulose, hemicellulose and lignin. Previous studies have already confirmed that cellulose is the responsible of the natural fibres mechanical strength; therefore, a higher cellulose content improves fibre resistance [7]. Flax fibre, together with sisal or hemp, has a high cellulose content, around 80%, so its mechanical properties are well considered as composites reinforcement [8]. 

The capital problem when using natural fibres as a reinforcement is their high water absorption due to their hydrophilic character, which lead in a weak interaction with the hydrophobic polymer matrix [9]. This problem is frequently solved by surface treatments that improve either mechanical or chemical anchoring [10]. Commonly, these are chemical treatments such as alkali or silane [11,12,13]. In order to reduce the waste generated during manufacturing by as much as possible, an environmentally friendly treatment is required. Plasma sources represent a green, fast, nontoxic and dry process which act on the surface material without affecting bulk properties [14,15], so it is presented as an important alternative for natural fibres in order to avoid other pollutant chemical treatments. In this research, Atmospheric Pressure Plasma Torch (APPT) was used over a unidirectional woven flax reinforcement. In previous works, the influence of Low Pressure Plasma (LPP) as a suitable treatment for natural short flax and coconut fibres with a random arrangement within a low density polyethylene (LDPE) matrix was evaluated. It was found to have a better adhesion between natural fibres and matrix as well as mechanical properties and thermal and moisture durability [16,17]. This effect is due to dehydration and cleaning of the fibres. This allows the carbonyl groups to have a better anchorage when the fibres are treated.

On the other hand, it would be necessary to use green polymers as a polymeric matrix. Thermoplastics, instead of thermosets, have been gaining importance due to their ability to be shaped repeatedly by melting them and for their recyclable character which allows an environmentally friendly material end of life [18].

Bioplastics, also called biopolymers, emerge as a solution to scarcity and, consequently, to the increasingly high price of petroleum. The new environmental regulations and the general awareness of respect for the environment promote the development of new materials and products considering the entire life cycle of them, from its obtaining, manufacturing, distribution, service life and end of life. In this way, a greater degree of independence from fossil fuels and a better waste management at the end of the useful life of each product can be achieved [19]. In this way, there are many applications of natural fibre/biopolymer composites in the industrial sector, especially in the automotive industry. Every year, end-of-life vehicles (ELV) generate between 7 and 8 million tonnes of waste in the European Union which have to be disposed of correctly. Interior parts of vehicles could be considered one of the main applications of these type of materials due to its ability to be recycled and to reduce the overall vehicle weight. There are many types of bioplastics. In this work, two bioplastics were used as matrices of flax fibre reinforced composites. Biobased polymers, such as GPE, are those obtained from natural resources instead of fossil fuels [20]. Polyolefins are the most remarkable polymers of this group. They are not biodegradable, but at the end of their useful life, they can be recycled, reshaped or disposed of by thermal decomposition. In GPE, the synthesis of the ethanol comes from sugar cane. Sugarcane captures CO_2_ from the atmosphere for the production of ethanol, from which ethylene is obtained. After a polymerisation process, ethylene will be converted to polyethylene [21].

**Figure 1:**
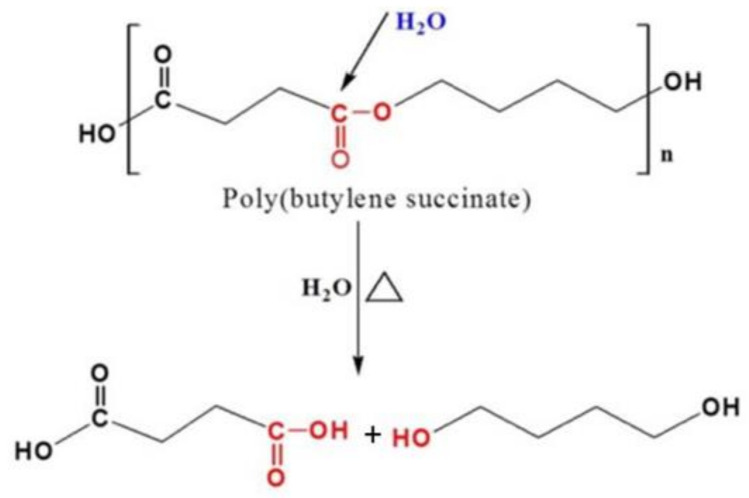
PBS hydrolysis reaction.

PBS is a synthetic biodegradable aliphatic polyester of petrochemical origin. It is obtained by combining 1,4-butanediol with succinic acid and its biodegradable characteristic is due to its tendency to hydrolyse, as it can be seen in Figure 1 [22,23].

Despite its fossil origin, 100% biodegradable composites can be obtained using PBS as matrix, natural fibres as reinforcement and plasma sources as surface treatment. PBS stands out for its excellent mechanical properties, its high melting point and its good processability.

**Figure 2:**
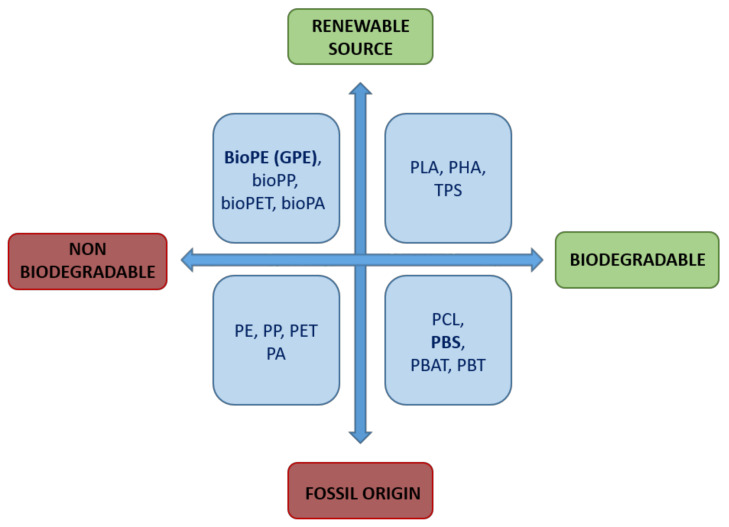
Types of biopolymers.

Finally, the last group of bioplastics are those biodegradables and whose origin come from a renewable source, such as polylactic acid (PLA), which is the most important and focus of numerous investigations [24,25]. Figure 2 shows a diagram of the types of bioplastics.

## 2. Materials and Methods 

### 2.1. Materials and Sample Preparation

Unidirectional woven flax Biotex 275 g/m^2^, was used as reinforcement of all composites and it was provided by Easy Composites (Staffordshire, UK). Composites were manufactured in a hot plates press, at 130 °C, with minimum pressure of the equipment 22.5 kN and during 13 min, by placing between two sheets of the corresponding matrix one layer of woven flax. During heating inside the hot plate press, polymers melt wetting the woven flax ensuring an intimate contact between them. The thickness of the final composite was delimited to 1 mm and the ratio reinforcement-matrix was around 30 wt% of flax fabric. Low density green polyethylene (GPE), SPB208, was provided by Braskem (San Paulo, Brazil) and polybutylene succinate (PBS) was supplied by TNJ Chemical Industry (Hefei, China). Both polymers were supplied in pellets form so sheets of each polymer had to be made also in the hot plate press. Samples were cut according to test from some sheets of composites. Both matrices were characterised in terms of surface energy, confirming the dispersive character of them.

### 2.2. APPT Treatment

An APPT device from PlasmaTreat GmbH (Steinhagen, Germany) was used to treat woven flax. The setup operated at a frequency of 17 kHz and a high-tension discharge of 20 kV. It was provided with a rotating torch ending in a nozzle (1900 rpm) through which plasma was expelled. The system contains an electronically speed controlled platform where the samples are placed. The air plasma was generated at a working pressure of 2 bar inside the rotating nozzle by non-equilibrium discharge and expelled through a circular orifice onto the samples. The speed of the platform was set at 2.5 m/min, and the distance between flax sample and the plasma nozzle was 20 mm.

### 2.3. Tensile Tests

Tensile tests of both polymeric matrices and composite materials were carried out on a universal electromechanical testing machine Microtest EM2/FR (Madrid, Spain) according to UNE-EN ISO 527-1:2012 [26]. Ten samples were tested for each material.

### 2.4. Peeling Tests

**Figure 3:**
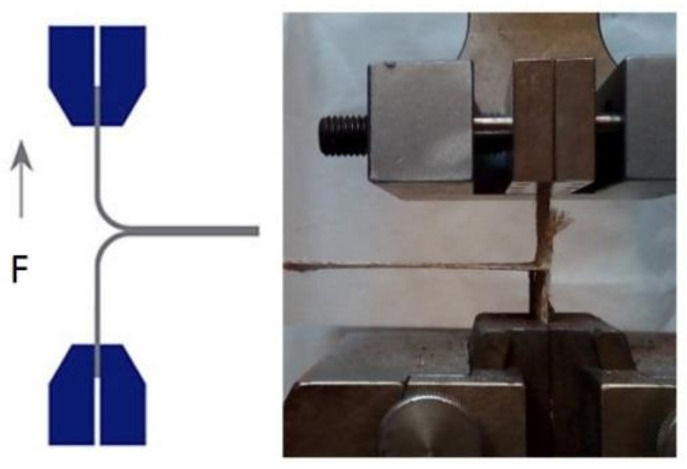
T peeling test layout.

T peeling tests were carried out according to UNE EN ISO 11339:2010 [27] standard in order to compare the peeling strength of APPT treated and untreated woven flax composites. Two layers of unidirectional woven flax were attached to each other by the matrix under study which acts as the adhesive between both flax layers and exerts the tensile force as indicated in Figure 3. The tests were carried out with a load cell of 1 kN and at a speed of 5 mm/min on a universal electromechanical testing machine Microtest EM2/FR (Madrid, Spain).

This test also provides information of the type of break that has occurred: cohesive, adhesive or mixed.

### 2.5. Moisture Absorption Tests

Both matrices and composites samples were placed in a furnace at 80 °C and relative humidity (RH) between 70 and 78%. Samples were placed inside a watertight container with water, on a grid so the specimens did not come into contact with water. The aging times analysed were 1, 2, 8, 30 and 60 days. Temperature and humidity conditions inside the container were controlled by means of a thermo-hygrometer (MSR 145 Electronics GmbH, Seuzach, Switzerland) throughout the aging period. After removing them from the oven, surfaces were dried with absorbent papers and weighed immediately. After each aging time, water absorption was evaluated by the relative uptake of weight, *M_t_*, according to Equation (1):

(1) $$M_{t}=\frac{W_{t}-W_{0}}{W_{0}}\times 100$$

where *W_0_* is the weight of dry specimen and *W_t_* is the weight of wet specimen at each aging time.

### 2.6. FTIR

Before and after aging conditions, matrices and composites were analysed with infrared spectroscopy (FTIR) to study the behaviour of all materials against water absorption. A Brucker Tensor 27 (Brucker Optik GmbH, Ettlingen, Germany) spectrometer was used to obtain the infrared spectra of all samples. The attenuated total multiple reflection technique (ATR) was used to analyse the surface chemical modifications produced up to about 5–10 mm deep into the pieces. A diamond prism was used and the incident angle of the IR radiation was 45°. Thirty-two scans with a resolution of 4 cm^−1^ were obtained and averaged. Spectra were recorded from 4000 to 650 cm^−1^. 

## 3. Results

### 3.1. Mechanical Properties

#### 3.1.1. Mechanical Properties of Matrixes

**Figure 4:**
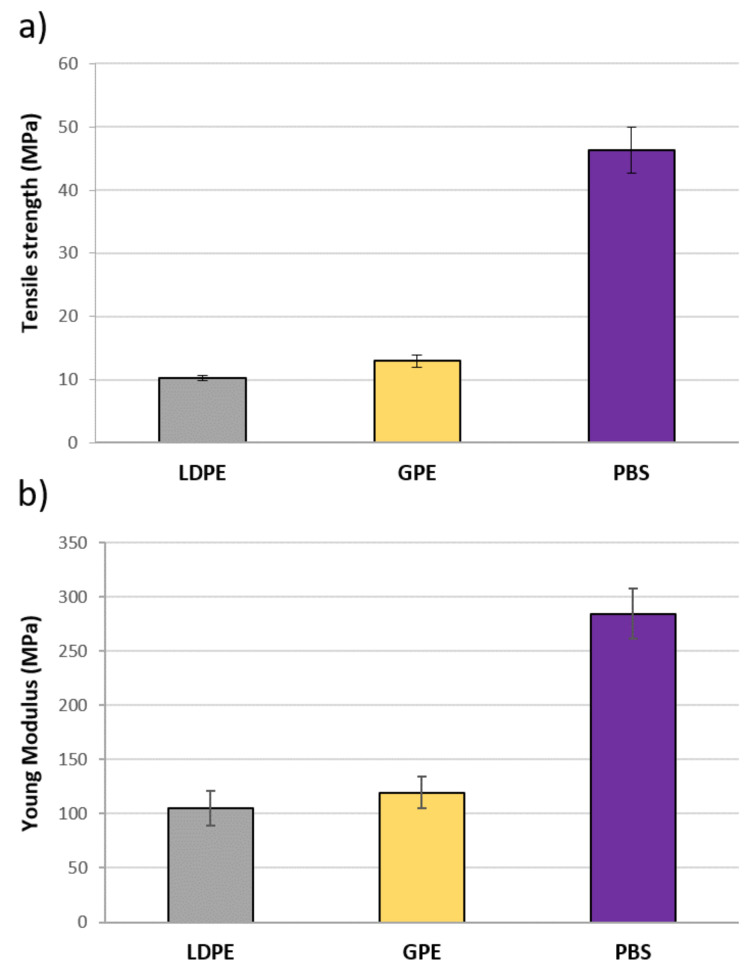
Mechanical properties of matrices. (**a**) Tensile strength and (**b**) Young’s modulus.

Tensile tests were carried out on both green polyethylene (GPE) and polybutylene succinate (PBS) to confirm the tensile strength provided by the suppliers and to compare GPE values with those obtained for another LDPE. As it can be observed in Figure 4, PBS has a considerably higher tensile strength and Young’s modulus than GPE, around 45 MPa against 12 MPa. If this is added to the fact that it is 100% biodegradable, PBS is presented as a good option to act as the matrix of composite materials reinforced with natural fibres. It can be also confirmed that GPE mechanical properties are very similar to those of any low density polyethylene, so it is also a good green alternative.

#### 3.1.2. Mechanical Properties of Composites

**Figure 5:**
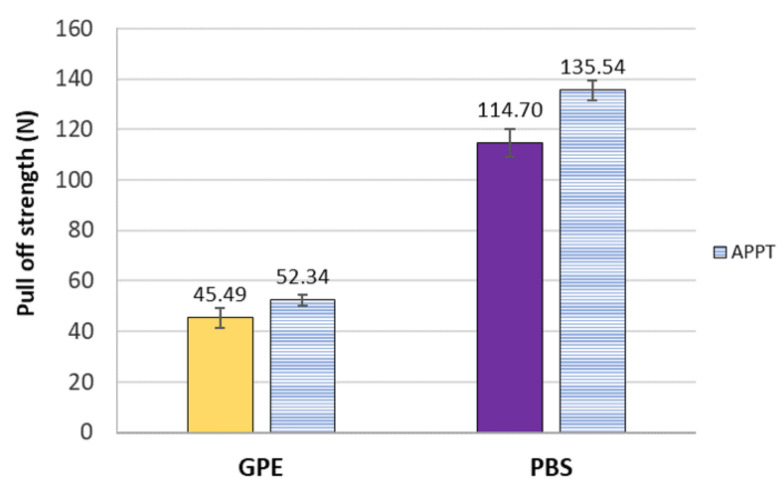
Peeling force between matrices and woven flax.

In order to obtain the adhesion force between each matrix and the woven flax, peeling tests were carried out as described in epigraph 2.4. APPT treatment effect was very significant by providing increments in peeling force of around 15–18% comparing untreated and APPT treated flax. Regarding Figure 5 can be also concluded that the adhesion PBS-flax is much better than GPE-flax.

**Figure 6:**
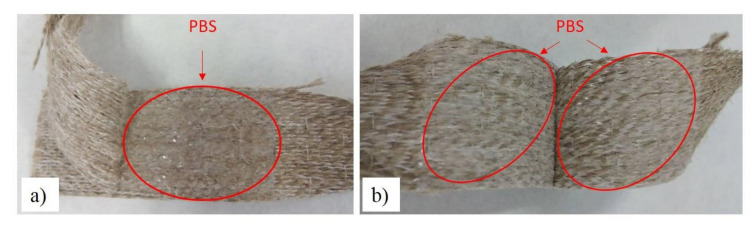
Failure types of PBS samples after peeling tests, (**a**) adhesive and (**b**) cohesive.

Another qualitative property that this test provides is the type of break that takes place after peeling tests, which gives an idea of how strong the bond is. In the case of APPT treated flax samples these failures were cohesive, with polymer sticking to each flax layer. In untreated materials, the failure was adhesive with polymer completely sticking only to one side. This behaviour is reflected in Figure 6 for PBS samples.

**Figure 7:**
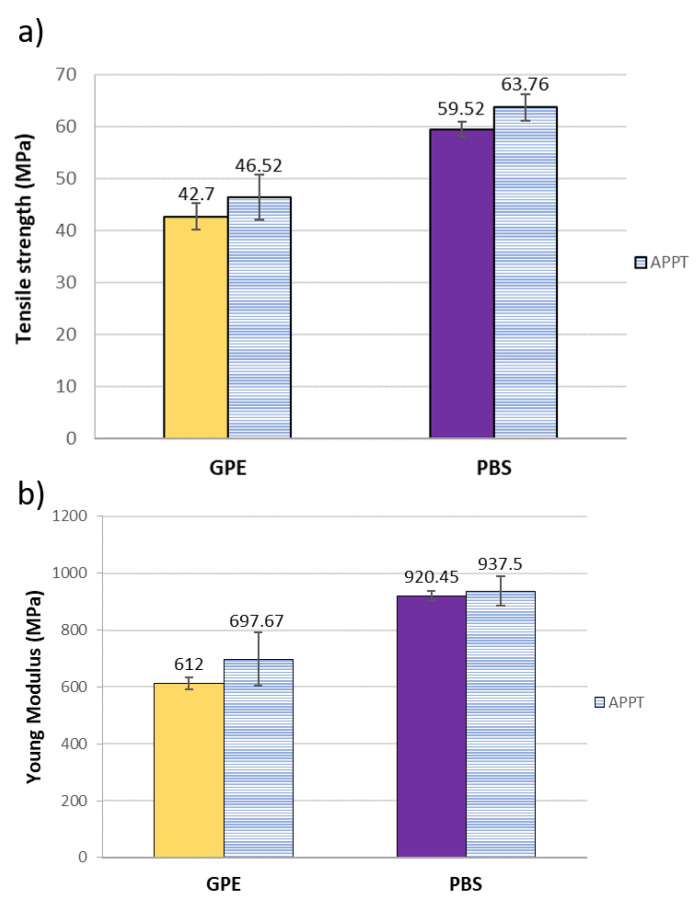
Mechanical properties of GPE and PBS composites. (**a**) Tensile strength and (**b**) Young’s modulus.

After manufacturing composite materials, tensile tests were performed with normalised specimens to obtain tensile strength and Young’s modulus of each composite and quantified the influence of APPT treatment in the final material (Figure 7).

As it can be seen, APPT treatment has improved mechanical properties of all composite but in a very slightly way. If these results are compared with those obtained for short flax fibre reinforced composites in previous works, it can be concluded that the effect of plasma treatment over short fibres becomes more visible than modifying unidirectional woven flax. This may be due to the industrial processes that are necessary to manufacture woven flax and not for obtaining short fibres. Fibres for unidirectional woven flax must be previously dried and washed to proceed with the weaving process, so the cleaning effect of plasma treatment is not as relevant as in short fibres [28]. 

On the other hand, the manufacture and structure of composite materials with unidirectional flax and short fibres is very different and its interaction with the matrix is also different. In the case of short fibres, their random distribution means that if the fibres are not well anchored to the matrix and there are gaps between them, they may resemble as defects in the material and therefore not achieve good properties of the final material. However, in the case of composites made with unidirectional fabric and manufactured by hot pressing, there is no such discontinuity, and the wetting of the fibres is easier. Nevertheless, APPT treatment improves mechanical properties of GPE and PBS composites in accordance with peeling tests. 

It is important also to note that the addition of the woven flax increase the tensile strength of GPE and PBS matrices in 75% and 37%, respectively.

### 3.2. Durability

#### 3.2.1. Moisture Absorption Tests

As it was previously mentioned, PBS is a 100% biodegradable polymer with a great capacity to be hydrolysed, so it can be expected that when it is subjected to adverse humidity and temperature conditions it will be deteriorated faster than GPE. 

**Figure 8:**
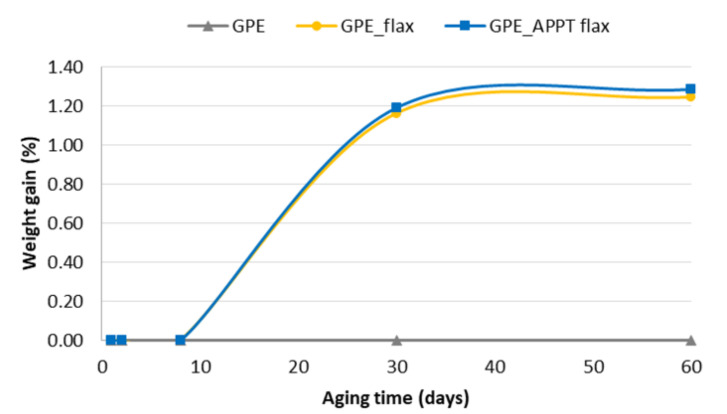
Weight gain of APPT treated and untreated GPE composites.

Weight gain measures of five samples of each GPE materials during aging time are shown in Figure 8. The error in these measurements is between ±0.1 and ±0.2. Regarding Figure 7 can be observed that unreinforced GPE practically shows no variation in its weight, so water absorption is zero. Polyolefins present a hydrophobic character and very good resistance against moisture, even at high temperatures [29].

However, GPE composites showed certain increase in weight after the 30 aging days, which was maintained until the end of the test. This value is not very significant because does not exceed 1.3% but reveals that flax fibres play an important role in durability due to their hydrophilic character. Although APPT treatment seems to have no effect, it is important to note that plasma treatment increases fibres wettability so it would be translated into a greater water absorption, but this effect does not occur due to the good adhesion between GPE and woven flax that prevents moisture absorption. 

**Figure 9:**
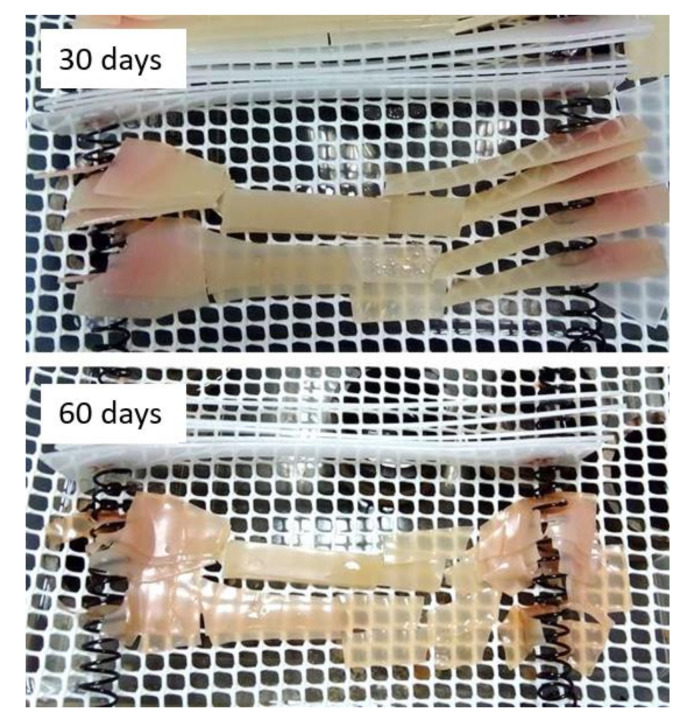
PBS samples under 30 and 60 aging days.

PBS behaviour is completely opposite. PBS matrix absorbs water from the first day until its total deterioration (Figure 9) preventing to evaluate the variations in weight beyond eight aging days. 

Although the weight variation was not excessive, 2.5%, but in line with that obtained by other authors [30], the deterioration of the material was visually evident, and 30 and 60 aging days mechanical properties could not either be evaluated.

The total degradation of the material is due to its biodegradable character caused by its hydrolysis capacity under conditions of high humidity and temperature. Typically, polymers with strong polar functional groups, such as amines or carbonyls, are capable of absorbing moisture through hydrogen bonds [31]. Therefore, it was expected that PBS, a polyester, can absorb more moisture than another practically dispersive polymer such as GPE, and polyolefins in general. It is common in polyesters that water will be accompanied by a mechanical properties loss [30,31].

**Figure 10:**
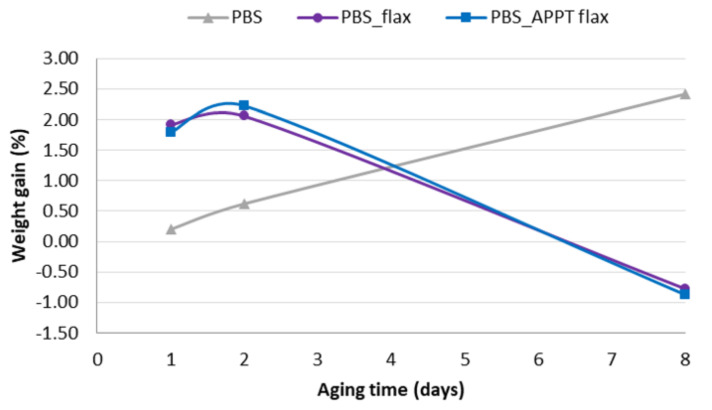
Weight gain of APPT treated and untreated PBS composites.

In Figure 10 can be observed that PBS composites absorb more moisture than PBS alone, mainly during the first days. This is due to the fact that in composite materials both woven flax and PBS absorbs water so the total absorption is greater. However, from eight aging days until the end of the test, the degradation of the material begins, due to the moisture already absorbed, and therefore its weight decreases slightly due to loss of material. After 30 days, PBS composites had completely lost their consistency.

Again, it is observed that APPT treatment does not negatively influence when the materials are subjected to high humidity environments and it fulfils its mission of improving the adhesion between flax and matrix preventing the penetration of moisture. Plasma treated fibres have more tendency to absorb moisture due to their dehydration during treatment and even considering that they are placed along the entire surface of the material. Since this effect does not happen, it means that the adhesion between woven fabric and PBS is good and does not allow to pass more moisture than without treatment.

#### 3.2.2. FTIR Analysis

The degradation of PBS and its composites as well as the low water absorption by GPE materials was confirmed by FTIR.

**Figure 11:**
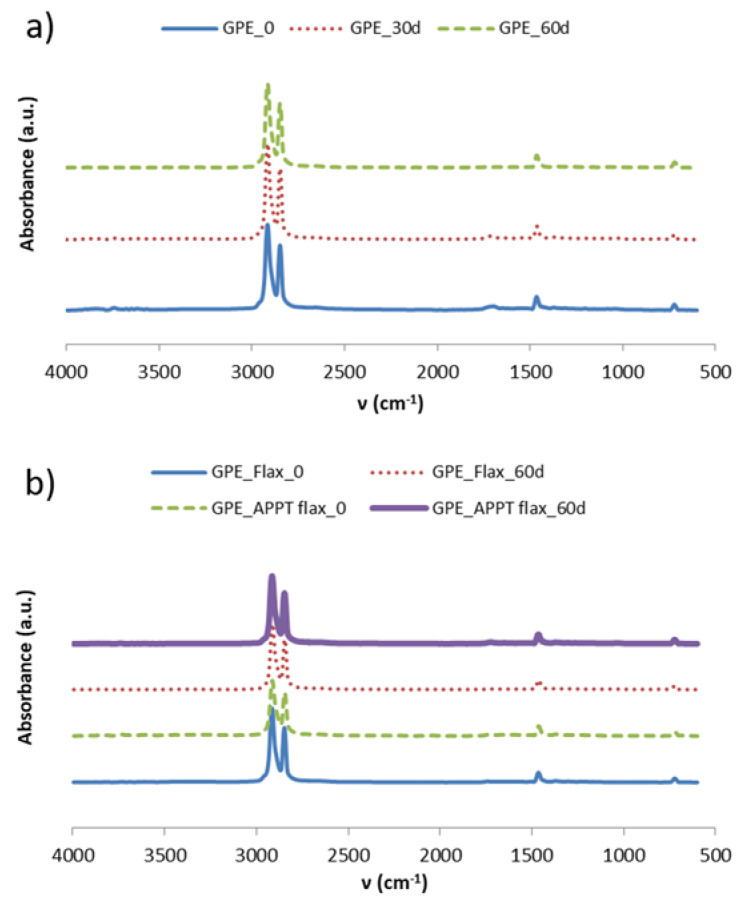
FTIR spectra for GPE materials before and after aging time. (**a**) GPE and (**b**) GPE composites.

Figure 11 shows the obtained spectra for GPE materials before and after the exposure time to high humidity and temperature. As it can be observed, both are practically the same and there are no variations in the water absorption band (3500 cm^−1^). There are also no differences between the APPT treated and untreated composite materials, so results from weight gain measurements are confirmed. GPE matrix was compared before aging and after 30 and 60 days, and the composites were compared before aging and only after 60 days, with and without APPT treatment. In composite materials, a small difference in the water absorption zone should be seen, since these increased their weight a 1.3%, but because of it is a small amount, it could be outside of the detection range of the equipment. 

The observed peaks correspond to the vibrations of the C–H bond. The band between 2800 and 3000 cm^−1^, is due to the CH and CH_2_ asymmetric stretching, around 1470 cm^−1^ appears the peak corresponding to the bending deformation of C–H bond and at 700 cm^−1^ take place the rocking deformation of C–H group. Another very small peak appears at around 1700 cm^−1^. This peak is related to the very sensitive ATR technique used and corresponds to the vibrations of amines originated by the UV stabilisers of the equipment [32].

**Figure 12:**
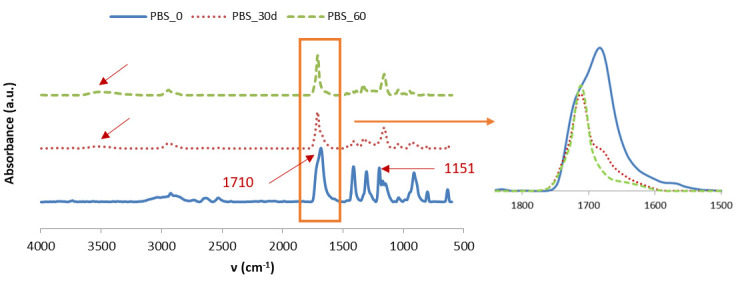
FTIR spectra for PBS before and after aging time.

Regarding PBS matrix, in Figure 12 it is observed that the peaks associated with the C=O bonds, at 1710 cm^−1^ and–COC– at 1151 cm^−1^, decrease due to a descent in molecular weight and deterioration of the chemical structure due to hydrolysis [33]. The variation of the peak at 1710 cm^−1^ is better appreciated when the three peaks of each aging time are overlapped as is shown in the right side of Figure 11. An increase in the water absorption band is also observed, around 3500 cm^−1^, which becomes more noticeable at 60 than at 30 days.

**Figure 13:**
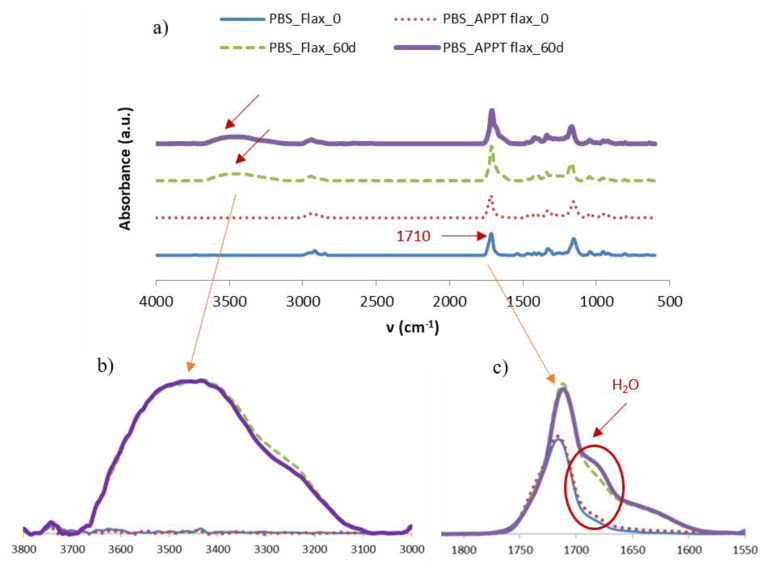
FTIR spectra for (**a**) PBS composites before and after aging time, (**b**) Detail of the overlapped spectra in the water absorption band between 3000 and 3500 cm^−1^ and (**c**) Detail of the overlapped spectra in the C=O absorption band between 1650 and 1750 cm^−^^1^.

In PBS composites, the main difference that can be observed is a considerable change in the water absorption band (3500 cm^−1^), which increases considerably after 60 days, as shown in Figure 13b. This absorbance is greater than the one obtained for the PBS alone, so it is verified that the presence of the woven flax contributes to the absorption of water. However, the band of the C=O bonds increases with aging, Figure 13c, when, due to hydrolysis, it should decrease as in the case of PBS. This occurs due to the presence of flax fibres, which, around 1700 cm^−1^, also have C=O bonds. Furthermore, between 1650 and 1700 cm^−1^ there is another band of water absorption that could overlap with the C=O peak, causing it to increase slightly and, specially, causing its widening.

Regarding the effect of the APPT treatment, no significant differences are observed and the absorption of the treated and untreated materials is practically the same. However, in Figure 13c, a small increase in the water absorption band is observed around 1675 cm^−1^, in the APPT treated composites after 60 days.

#### 3.2.3. Mechanical Properties

Mechanical properties are the primary indicators for evaluating the durability of polymeric materials. The influence of temperature and humidity on the mechanical properties, as well as the effect of the APPT treatment, were evaluated by tensile tests.

**Figure 14:**
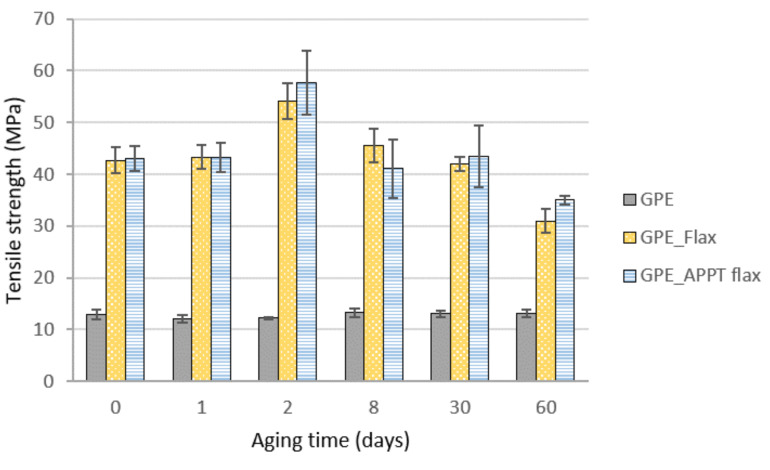
Tensile strength of GPE materials during aging time.

Due to the high resistance of the GPE against humidity, tensile strength values of both composites and GPE matrix, remained practically unchanged until 30 aging days. Only it is observed a little descent of resistance after 60 days, around a 16%, which coincides with the maximum moisture absorption by the materials, as shown in Figure 14. For long aging times, tensile strength is less affected in APPT treated composites, demonstrating that the better adhesion improved by the plasma treatment is more durable than in untreated materials. After two days, composites tensile strength increases considerably, which can be attributed to an increase in crystallinity due to temperature and humidity [30].

**Figure 15:**
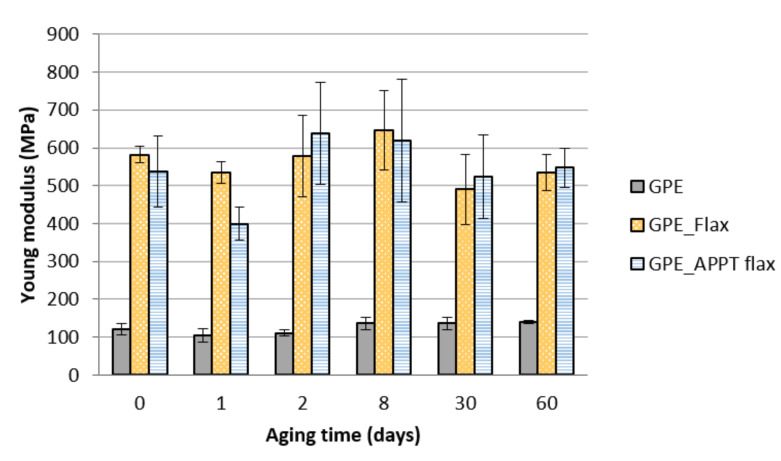
Young’s modulus of GPE materials during aging time.

The unreinforced GPE practically did not suffer any variation. Although humidity does not have a noticeable influence, as it was previously mentioned, the high temperature should have increased its resistance, since it favours the mobility of the chains, increasing their crystallinity. Young’s modulus evolution is shown in Figure 15. GPE did not either undergo any variation, as well as composites that practically maintain Young’s modulus values obtained before aging.

Accordingly, it can be concluded that GPE materials are not highly affected by aging conditions from the point of view of mechanical behaviour. 

**Figure 16:**
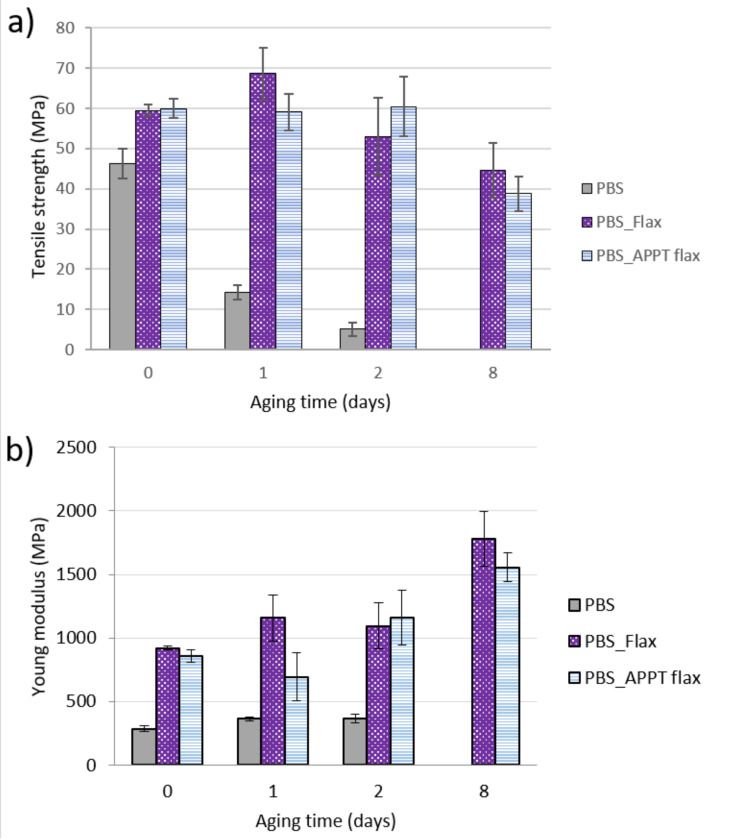
Mechanical properties of PBS materials during aging time. (**a**) Tensile strength and (**b**) Young’s modulus.

Figure 16 shows mechanical behaviour of PBS and its composite materials. The most remarkable is the great difference between the behaviour of PBS and PBS reinforced with woven flax. PBS, after 1 day under aging conditions, reduces its tensile strength from 46 MPa to 14 MPa and it continues decreasing up to two days. On the eighth day, no tensile tests could be carried out since the specimens broke just by taking them or placing them in the testing machine. This decrease in resistance values can be attributed to a combined effect between degradation by hydrolysis and reduction in molecular weight as a consequence of aging conditions [34]. Young’s modulus practically did not vary, although it can be considered a slight rise, which would increase the stiffness of the PBS.

PBS composites did not lose resistance until the eighth day, indicating that despite the fact that the water absorption by these materials is greater than the water absorption by PBS alone, flax fibres hinder or delay the hydrolysis process that the matrix suffer. Young’s modulus of composites increased with aging, being more noticeable after eight days. Therefore, after eight aging days, PBS composites become stiffer and less resistant. After 30 days, PBS composites were completely degraded, thus mechanical properties could not be evaluated.

## 4. Conclusions

In the present work, two types of green polymer reinforced with woven flax were compared and studied from the point of view of mechanical properties and durability under aging conditions. The influence of APPT treatment was also studied. With the aim of obtaining a 100% biodegradable or recyclable composite, with good mechanical properties that allow it to be used under controlled adverse conditions. 

First of all, PBS presents higher tensile strength than GPE, and in addition is biodegradable so it is presented as a good alternative for green composite materials.

As shown by peeling tests, the adhesion between woven flax and PBS is much greater than the one between flax and GPE. In both cases the peeling force in improved by APPT treatment, achieving a better matrix-fibre interaction.

Mechanical properties of composites showed a slight improvement with APPT treatment. However, the greater adhesion between flax and matrices was demonstrated by practically the same moisture absorption from APPT treated and untreated materials.

It can be also concluded that GPE composites have a greater durability, against adverse temperature and humidity conditions, than PBS materials, due to the tendency of polyester to hydrolyse compared to the good resistance against moisture of polyolefins. In both GPE and PBS composites, better mechanical properties were obtained, when reinforcing with unidirectional woven flax, both before and after aging conditions.

Consequently, flax reinforcement is a good and green option for composites as well as the two studied matrices. PBS composites are 100% biodegradables, with excellent mechanical properties and less durability. Conversely, GPE composites are not biodegradable, but they are recyclable and have a very good durability and somewhat lower mechanical properties.
